# Successful Growth Hormone Therapy in Cornelia de Lange Syndrome

**DOI:** 10.4274/jcrpe.4349

**Published:** 2017-12-15

**Authors:** Michael de Graaf, Sarina G Kant, Jan Maarten Wit, Egbert Johan Willem Redeker, Gijs Willem Eduard Santen, Annemieke Johanna Maria Henriëtta Verkerk, André Gerardus Uitterlinden, Monique Losekoot, Wilma Oostdijk

**Affiliations:** 1 Leiden University Medical Center, Department of Pediatrics, Leiden, The Netherlands; 2 Leiden University Medical Center, Department of Clinical Genetics, Leiden, The Netherlands; 3 Academic Medical Center, Department of Clinical Genetics, Amsterdam, The Netherlands; 4 Erasmus Medical Center, Department of Internal Medicine, Rotterdam, The Netherlands

**Keywords:** Cornelia de Lange syndrome, growth hormone, small for gestational age, NIPBL, whole-exome sequencing

## Abstract

Cornelia de Lange syndrome (CdLS) is a both clinically and genetically heterogeneous syndrome. In its classical form, it is characterised by distinctive facial features, intra-uterine growth retardation, short stature, developmental delay, and anomalies in multiple organ systems. NIPBL, SMC1A, SMC3, RAD21 and HDAC8, all involved in the cohesin pathway, have been identified to cause CdLS. Growth hormone (GH) secretion has been reported as normal, and to our knowledge, there are no reports on the effect of recombinant human GH treatment in CdLS patients. We present a patient born small for gestational age with persistent severe growth retardation [height -3.4 standard deviation score (SDS)] and mild dysmorphic features, who was treated with GH from 4.3 years of age onward and was diagnosed 6 years later with CdLS using whole-exome sequencing. Treatment led to a height gain of 1.6 SDS over 8 years. Treatment was interrupted shortly due to high serum insulin-like growth factor-1 serum values. In conclusion, GH therapy may be effective and safe for short children with CdLS.

**What is already known on this topic?**

This manuscript describes a patient with Cornelia de Lange syndrome who was successfully treated with growth hormone because she was born small for gestational age and showed no catch up growth. The diagnosis of Cornelia de Lange syndrome was made years after the growth hormone treatment. To our knowledge, there is no published work about the effect of growth hormone therapy in Cornelia de Lange syndrome.

**What this study adds?**

This case report will contribute to more knowledge about treatment with growth hormone in Cornelia de Lange syndrome and also help fellow pediatricians and pediatric endocrinologists in their decision-making when considering the treatment in this patient group.

## INTRODUCTION

The clinical features of Cornelia de Lange syndrome (CdLS) (synonym Brachmann-de Lange syndrome, de Lange syndrome; Online Mendelian Inheritance in Man #122470, #300590, #610759, #614701, #300882) were first described by de Lange (1) in 1933, although Brachmann (2) is believed to have reported a case in 1916. CdLS is known as a rare and genetically and clinically heterogeneous disorder. The reported prevalence of 0.5-10:100,000 may be an underestimation due to underdiagnosis of mild cases (3).

Mutations in the NIPBL, SMC1A, SMC3, RAD21, and HDAC8 genes have been identified to cause CdLS (4). These genes code for subunits and regulatory proteins in the cohesin pathway. Most cases of CdLS are sporadic although autosomal and X-linked inheritance patterns have been described in some families (4).

CdLS is a disorder affecting multiple organ systems. Characteristic craniofacial features include well-defined arched eyebrows with synophrys, long and curly eyelashes, ptosis and low-set, posteriorly rotated ears. In addition, a variety of additional features such as craniofacial deviations and cardiovascular, gastrointestinal, genitourinary, and neurosensory abnormalities have been reported. Skeletal deformations usually affect the upper extremity. Linear growth is in general impaired, and affected patients show both pre- and postnatal growth retardation. CdLS specific growth charts are available showing a mean adult height of 155.8 cm for males and 131.1 cm for females (5). Cognitive and psychomotor abilities vary from mild learning disabilities, in which speech and language disorders are more distinct, to severe intellectual disability (4). With regard to the severity of clinical features, there is a trend of a genotype-phenotype association, depending on the causative gene and type of mutation. However, an identical mutation can be associated with quite different phenotypes, suggestive of a role of other modifying genetic or environmental factors (6).

Only few reports have commented on endocrine abnormalities in CdLS. Kline et al (7) reported a mildly delayed puberty in a group of 49 patients with CdLS, with a mean age of onset of 15 years for boys and 13 years for girls. In several reports, growth hormone (GH) secretion was assessed and found normal. We found no reports on the effect of recombinant human GH (r-hGH) treatment in children with CdLS.

We present a female patient born small for gestational age (SGA) with severe postnatal growth retardation and mild dysmorphic features. She was treated with GH from 4.3 years of age and was diagnosed 6 years later with CdLS using whole-exome sequencing (WES), thereby being the first reported CdLS patient to be treated with GH.

## CASE REPORTS

**Figure 1:**
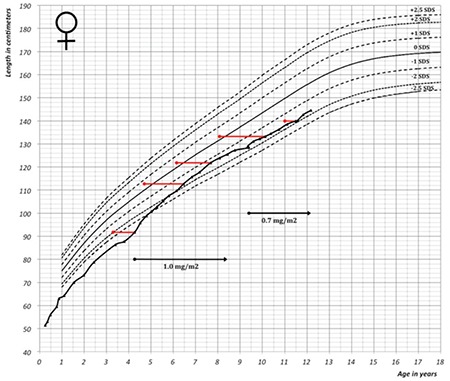
Growth curve of the patient plotted on the growth reference chart for female Dutch children (-2.5, -2.0, -1.0, 0, +1.0, +20 and +2.5 standard deviation lines are shown). Red lines indicate bone age, horizontal lines indicate recombinant human growth hormone treatment (8) SDS: standard deviation score

This female patient is the third child of healthy non-consanguineous parents with normal heights (father 173 cm, mother 166 cm) and no known familiar diseases or genetic defects. Conditional target height (cTH) was 166 cm [-0.7 standard deviation score (SDS)] (8). Both her siblings had normal birth weight and postnatal growth. At the age of 10 months, she was referred to our centre for evaluation of short stature. After being carefully monitored in the prenatal period for intra-uterine growth retardation, she was born at a gestational age of 37 weeks and 5 days, after an uncomplicated delivery. At birth, her weight was 2340 grams (-1.7 SDS) (9), but subsequent measurements of weight in the first year of life were between -3.4 SDS and -4.0 SDS. Head circumference at birth was 32 cm (-2.4 SDS). Length was not measured at birth, but at 3 months of age length was 51.5 cm (-3.4 SDS), and all subsequent height measurements were below -3.0 SDS (Figure 1). Based on these data, we estimated the likelihood of a low birth length sufficient to diagnose the child as SGA with failure of catch-up growth (10).

At the age of 10 months, length was 63 cm (-3.7 SDS), weight 6.2 kg (-3.4 SDS), weight for length was normal (0.2 SDS), head circumference was 42.5 cm (-2.0 SD), and the ratio between crown-rump length and total length was 0.68 (2.1 SDS) (11). Arm span/height ratio was 0.92 (0.1 SDS) (12). Physical examination showed no abnormalities except for two dysmorphic features. First, the patient had well-defined and arched eyebrows with synophrys. Second, she had short digits of both hands, in particular the phalanges of both fifth digits (Figure 2a, 2b).

Serum insulin-like growth factor-1 (IGF-1) was 8.0 nmol/L (-0.1 SDS), IGF-binding protein 3 1.8 mg/L (-1.1 SDS), and GH stimulation tests with clonidine and arginine showed serum GH of peaks of 4.4 μg/L and 12.1 μg/L, respectively. This made GH deficiency or insensitivity unlikely.

A skeletal survey performed in the first year of life and at the age of two years showed no signs of skeletal dysplasia. A 46,XX karyotype was determined at amniocentesis, and genetic evaluation of SHOX, FGFR3, IGF-1, and IGF1R revealed no mutations or copy number variants, and uniparental disomy of chromosome 7 and hypomethylation of the 11p15 imprinting region tested negative. At the age of 5 years, a single nucleotide polymorphism array (GeneChip Human Mapping 250K Nsp Array) was performed showing no abnormalities.

At the age of 10 years, WES was performed in the index patient and her parents. Genomic DNA was isolated from peripheral blood samples using the Autopure LS Instrument (Gentra Systems). Cytogenetic microarray analysis was performed using the Affymetrix CytoScan HD Array according to the manufacturer’s procedures. Copy number was assessed in the patient using ChAS software (Chromosome Analysis Suite). WES was performed as previously described by Hannema et al (13), using the Nimblegen SEqCap EZ V2 capture kit (Roche, Nimblegen, Inc, Madison, Wisconsin). Filtering for de novo variants in the index patient with a home-made variant filtering algorithm (Santen et al) (14) showed a heterozygous, de novo splice mutation (c.771+1G>A), chr5:36971139 build 37, in NIPBL gene. Although this mutation has not been described previously, it has been found in another patient with CdLS (15). The result was confirmed by Sanger sequencing. cDNA analysis was performed to investigate the effect of the splice mutation. An aberrant transcript besides the expected wild type PCR product was observed. Sequencing of the aberrantly spliced product revealed a loss of the last 21 bp of exon 7, which is expected to lead to an in-frame deletion of 7 amino acids in the protein, p.(Val251_Asp257del).

Following the diagnosis of CdLS, an extensive medical screening was performed. Echocardiography revealed a structurally and functionally normal heart. An ultrasound of the urogenital tract showed normal structures. Otorhinolaryngologic and ophthalmologic evaluations showed no abnormalities except a mild nervus IV paresis of the left eye, but without resulting in a negative effect on the visual performance of the child.

**Figure 3:**
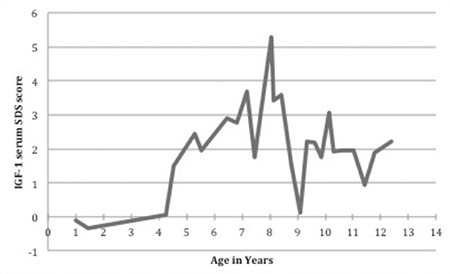
Patient’s serum insulin-like growth factor-1 standard deviation score IGF-1: insulin-like growth factor-1, SDS: standard deviation score

Treatment with r-hGH in a dosage of 0.86 mg/m2/day subcutaneously was started at 4.3 years of age at a height of 91.7 cm (-3.5 SDS) based on the indication of SGA with failure of catch-up growth. Bone age, assessed according to the Greulich and Pyle (16) method, was then 3.3 years. After the initiation of GH treatment regular outpatient clinic visits were scheduled. Every three months, anthropometric values were evaluated. Bone age and laboratory tests including IGF-1 were performed yearly. The growth response was appropriate (Figure 1) and at 8.5 years of age, height was 125.5 cm (-1.4 SDS). Following a series of increased serum IGF-1 values, as high as 5.3 SDS (Figure 3), therapy was interrupted for nine months, which led to deceleration of growth (height -1.7 SDS). At 9.3 years, r-hGH therapy was restarted at a lower dose of 0.7 mg/m2. At present, at the age of 12.3 years, her height is 142.6 cm (-1.8 SDS) and IGF-1 is 2.2 SDS. The last measured bone age was 11.0 years at an age of 11.6 years. Predicted adult height at that point according to Bayley and Pinneau (17) was 154.4 cm, 1.8 SD below cTH-SDS. No side effects or health complaints occurred during therapy. Due to a speech and language disorder, she followed an adjusted educational programme. Her intellectual abilities were tested within the normal range with a nonverbal intelligence quotient of 98. Motor development was normal.

The patient’s parents gave their consent for inclusion of the patient’s pictures in this publication.

## DISCUSSION

In this case report, we present a female patient with extreme short stature and very mild clinical features of CdLS. She showed a substantial increase in height SDS of 1.8 SDS following treatment with r-hGH. To our knowledge, this is the first report on r-hGH treatment in a patient with CdLS. SGA without adequate catch-up growth is an established indication for GH therapy in most parts of the world including Europe and for this reason, GH was started in our patient. The described effect of r-hGH treatment in our patient resembles the increase in height previously reported in SGA patients treated with a similar GH dose (18,19). The initial extreme increase in IGF-1 SDS score on the 0.86 mg/m^2^ GH dose is considerably higher than that reported for GH-treated children with SGA (18,20).

In approximately 70% of CdLS patients, a genetic cause is detected (4). A NIPBL mutation is found in 80% of these cases and results in more severe clinical problems in comparison to the other genes. However, missense mutations in this gene tend to give rise to a milder phenotype than splice site mutations, or nonsense and frameshift mutations (4). Our patient has a splice site mutation in the NIPBL gene, demonstrated by both DNA and RNA analysis, causing an in-frame deletion which resulted in mild clinical features. The most prominent clinical feature in this patient was growth retardation. Facial features of CdLS were not distinctive. Although she needed a special educational program for her speech language disorder, intelligence was within the normal range.

Although both prenatal and postnatal growth retardation is a hallmark of CdLS, little is known about CdLS and GH function. Schwartz et al (21), McArthur and Edwards (22), and Abraham and Russell (23) published data on GH concentration in 16 CdLS patients (see Table 1). Two patients reported by Schwartz et al (21) were diagnosed with classic GH deficiency. One of these well-nourished patient had low serum IGF-1 values and discordant results on stimulation tests, resulting in the conclusion that there was some degree of end-organ resistance to GH. McArthur and Edwards (22) and Abraham and Russell (23) measured GH concentration in 11 patients using multiple methods. GH concentrations of all patients were ≥14 μg/L.

In a more recent case series of 49 CdLS patients, 98 percent of the patients had heights below the 5th percentile compared to standard growth curves, but no tests for GH were conducted (7). Our patient had both normal GH concentrations after stimulation tests with arginine and clonidine as well as normal IGF-1 serum values, thereby excluding GH deficiency or insensitivity. The very high serum IGF-1 concentrations on a regular GH dosage suggest a form of IGF-1 insensitivity.

Although this case report suggests an appropriate growth response to r-hGH in this patient, we acknowledge that she has not achieved adult height yet. Still, our observation suggests that short children with CdLS (mildly affected) may be considered as candidates for r-hGH treatment.

## Figures and Tables

**Figure 2a:**
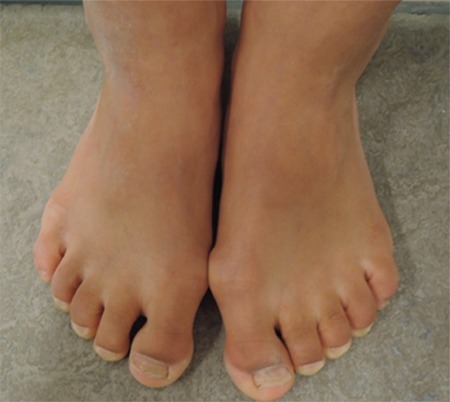
Images of the hands a showing short digits. Published with permission of the patient’s parents. No permission was given to publish photographs of facial features

**Figure 2b:**
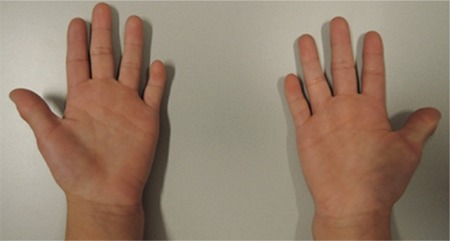
Images of the hands showing short digits, especially of the thumbs and fifth digits. Published with permission of the patient’s parents

**Table 1:**
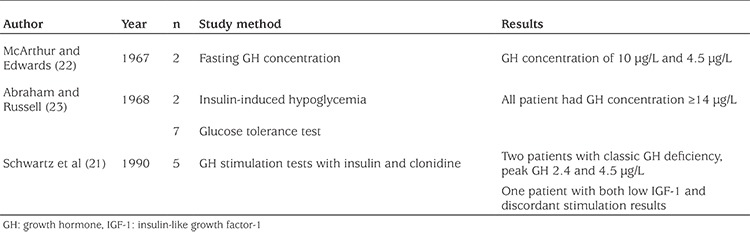
Overview of growth hormone test results in Cornelia de Lange syndrome patient

## References

[1] de Lange C (1933). Sur un type nouveau de degenerescence (typus Amstelodamensis). Arch Med Enfants.

[2] Brachmann W (1916). Ein fall von symmetrischer monodaktylie durch Ulnadefekt, mit symmetrischer flughautbildung, in den ellebeugen, sowie anderen abnormitaten (zwerghaftogkeit, halsrippen, behaarung). Jarb Kinder Phys Erzie.

[3] Van Allen MI, Filippi G, Siegel-Bartelt J, Yong SL, McGillivray B, Zuker RM, Smith CR, Magee JF, Ritchie S, Toi A, et al (1993). Clinical variability within Brachmann-de Lange syndrome: a proposed classification system. Am J Med Genet.

[4] Boyle MI, Jespersgaard C, Brondum-Nielsen K, Bisgaard AM, Tümer Z (2015). Cornelia de Lange syndrome. Clin Genet.

[5] Kline AD, Barr M, Jackson LG (1993). Growth Manifestations in the Brachmann-Delange Syndrome. Am J Med Genet.

[6] Mannini L, Cucco F, Quarantotti V, Krantz ID, Musio A (2013). Mutation spectrum and genotype-phenotype correlation in Cornelia de Lange syndrome. Hum Mutat.

[7] Kline AD, Grados M, Sponseller P, Levy HP, Blagowidow N, Schoedel C, Rampolla J, Clemens DK, Krantz I, Kimball A, Pichard C, Tuchman C (2007). Natural history of aging in Cornelia de Lange syndrome. Am J Med Genet C Semin Med Genet.

[8] van Dommelen P, Schönbeck Y, van Buuren S (2012). A simple calculation of the target height. Arch Dis Child.

[9] Niklasson A, Albertsson-Wikland K (2008). Continuous growth reference from 24th week of gestation to 24 months by gender. BMC Pediatr.

[10] Wit JM, Ranke BM, Kelnar CJH (2007). ESPE Classification of Paediatric Endocrine Diagnoses. Karger.

[11] Fredriks AM, van Buuren S, van Heel WJ, Dijkman-Neerincx RH, Verloove-Vanhorick SP, Wit JM (2005). Nationwide age references for sitting height, leg length, and sitting height/height ratio, and their diagnostic value for disproportionate growth disorders. Arch Dis Child.

[12] Gerver WJM, de Bruin R (1996). Paediatric Morphometrics a reference manual. Utrecht Bunge.

[13] Hannema SE, van Duyvenvoorde HA, Premsler T, Yang RB, Mueller TD, Gassner B, Oberwinkler H, Roelfsema F, Santen GW, Prickett T, Kant SG, Verkerk AJ, Uitterlinden AG, Espiner E, Ruivenkamp CA, Oostdijk W, Pereira AM, Losekoot M, Kuhn M, Wit JM (2013). An activating mutation in the kinase homology domain of the natriuretic peptide receptor-2 causes extremely tall stature without skeletal deformities. J Clin Endocrinol Metab.

[14] Santen GW, Aten E, Sun Y, Almomani R, Gilissen C, Nielsen M, Kant SG, Snoeck IN, Peeters EA, Hilhorst-Hofstee Y, Wessels MW, den Hollander NS, Ruivenkamp CA, van Ommen GJ, Breuning MH, den Dunnen JT, van Haeringen A, Kriek M (2012). Mutations in SWI/SNF chromatin remodeling complex gene ARID1B cause Coffin-Siris syndrome. Nat Genet.

[15] Yuan B, Pehlivan D, Karaca E, Patel N, Charng WL, Gambin T, Gonzaga-Jauregui C, Sutton VR, Yesil G, Bozdogan ST, Tos T, Koparir A, Koparir E, Beck CR, Gu S, Aslan H, Yuregir OO, Al Rubeaan K, Alnaqeb D, Bayram Y, Atik MM, Aydin H, Geckinli BB, Seven M, Ulucan H, Fenercioglu E, Ozen M, Jhangiani S, Muzny DM, Boerwinkle E, Tuysuz B, Alkuraya FS, Gibbs RA, Lupski JR (2015). Global transcriptional disturbances underlie Cornelia de Lange syndrome and related phenotypes. J Clin Invest.

[16] Greulich W, Pyle S (1959). Radiograph Atlas of Skeletal Development of the Hand and Wrist. 2nd ed.

[17] Bayley N, Pinneau SR (1952). Tables for predicting adult height from skeletal age: revised for use with the Greulich-Pyle hand standards. J Pediatr.

[18] Hokken-Koelega A, van Pareren Y, Arends N, Boonstra V (2004). Efficacy and Safety of Long-Term Continuous Growth Hormone Treatment of Children Born Small for Gestational Age. Horm Res.

[19] Van Pareren Y, Mulder P, Houdijk M, Jansen M, Reeser M, Hokken-Koelega A (2003). Adult height after long-term, continuous growth hormone (GH) treatment in short children born small for gestational age: results of a randomized, double-blind, dose-response GH trial. J Clin Endocrinol Metab.

[20] Tanaka T, Yokoya S, Seino Y, Togari H, Mishina J, Kappelgaard AM, Fujieda K (2011). Long-term efficacy and safety of two doses of growth hormone in short japanese children born small for gestational age. Horm Res Paediatr.

[21] Schwartz ID, Schwartz KJ, Kousseff BG, Bercu BB, Root AW (1990). Endocrinopathies in Cornelia de Lange syndrome. J Pediatr.

[22] McArthur R, Edwards JH (1967). De Lange syndrome: report of 20 cases. Can Med Assoc J.

[23] Abraham JM, Russell A (1968). De lange syndrome. A study of nine examples. Acta Paediat Scand.

